# Supplementary material for: Heparin-based hydrogel scaffolding alters the transcriptomic profile and increases the chemoresistance of MDA-MB-231 triple-negative breast cancer cells
Source: Biomater Sci. 2020 Feb 13;8(10):2786–96. doi: 10.1039/c9bm01481k (PMC7497406; doi:10.1039/c9bm01481k)
Supplement: Supplementary file 2 [file BM-008-C9BM01481K-s002.zip › Supplementary File 4/HP-BhydrogelvControl/Pathways/my_analysis.Gsea.1545201079907/HALLMARK_INTERFERON_ALPHA_RESPONSE.html]

Details for gene set HALLMARK\_INTERFERON\_ALPHA\_RESPONSE[GSEA]

|  || Dataset | expr.class.cls#HYD\_versus\_CONTROL.class.cls#HYD\_versus\_CONTROL\_repos |
| Phenotype | class.cls#HYD\_versus\_CONTROL\_repos |
| Upregulated in class | CONTROL |
| GeneSet | HALLMARK\_INTERFERON\_ALPHA\_RESPONSE |
| Enrichment Score (ES) | -0.3831313 |
| Normalized Enrichment Score (NES) | -1.6384027 |
| Nominal p-value | 0.0 |
| FDR q-value | 0.006255171 |
| FWER p-Value | 0.031 |
Table: GSEA Results Summary

  

Fig 1: Enrichment plot: HALLMARK\_INTERFERON\_ALPHA\_RESPONSE      
 Profile of the Running ES Score & Positions of GeneSet Members on the Rank Ordered List

  

| PROBE | DESCRIPTION (from dataset) | GENE SYMBOL | GENE\_TITLE | RANK IN GENE LIST | RANK METRIC SCORE | RUNNING ES | CORE ENRICHMENT || 1 | MOV10 | na |  |  | 721 | 2.097 | -0.0034 | No |
| 2 | RNF31 | na |  |  | 1752 | 1.469 | -0.0333 | No |
| 3 | PARP12 | na |  |  | 1935 | 1.394 | -0.0200 | No |
| 4 | OGFR | na |  |  | 2536 | 1.162 | -0.0324 | No |
| 5 | TRIM26 | na |  |  | 2685 | 1.122 | -0.0218 | No |
| 6 | IRF7 | na |  |  | 3003 | 1.036 | -0.0214 | No |
| 7 | TRIM14 | na |  |  | 4153 | 0.778 | -0.0688 | No |
| 8 | STAT2 | na |  |  | 4243 | 0.764 | -0.0610 | No |
| 9 | OAS1 | na |  |  | 4352 | 0.741 | -0.0545 | No |
| 10 | ISG15 | na |  |  | 4916 | 0.639 | -0.0735 | No |
| 11 | IFI30 | na |  |  | 5258 | 0.592 | -0.0817 | No |
| 12 | MX1 | na |  |  | 5402 | 0.570 | -0.0798 | No |
| 13 | IRF1 | na |  |  | 5648 | 0.533 | -0.0839 | No |
| 14 | OASL | na |  |  | 6099 | 0.470 | -0.0998 | No |
| 15 | SLC25A28 | na |  |  | 6103 | 0.470 | -0.0923 | No |
| 16 | UBA7 | na |  |  | 6122 | 0.468 | -0.0855 | No |
| 17 | LPAR6 | na |  |  | 6276 | 0.447 | -0.0862 | No |
| 18 | HERC6 | na |  |  | 6366 | 0.434 | -0.0838 | No |
| 19 | TRIM25 | na |  |  | 6494 | 0.416 | -0.0836 | No |
| 20 | SP110 | na |  |  | 6691 | 0.394 | -0.0874 | No |
| 21 | CXCL10 | na |  |  | 6890 | 0.372 | -0.0917 | No |
| 22 | DHX58 | na |  |  | 7492 | 0.309 | -0.1181 | No |
| 23 | IL7 | na |  |  | 8303 | 0.219 | -0.1569 | No |
| 24 | C1S | na |  |  | 8670 | 0.184 | -0.1731 | No |
| 25 | ADAR | na |  |  | 8794 | 0.167 | -0.1768 | No |
| 26 | IL4R | na |  |  | 9138 | 0.135 | -0.1925 | No |
| 27 | LAMP3 | na |  |  | 9393 | 0.105 | -0.2041 | No |
| 28 | CMPK2 | na |  |  | 10261 | 0.015 | -0.2492 | No |
| 29 | IFI44L | na |  |  | 10300 | 0.015 | -0.2510 | No |
| 30 | CASP1 | na |  |  | 10302 | 0.015 | -0.2508 | No |
| 31 | CNP | na |  |  | 10450 | 0.001 | -0.2584 | No |
| 32 | IFI35 | na |  |  | 10456 | 0.000 | -0.2587 | No |
| 33 | TAP1 | na |  |  | 10646 | -0.022 | -0.2682 | No |
| 34 | PARP14 | na |  |  | 10807 | -0.039 | -0.2760 | No |
| 35 | CASP8 | na |  |  | 10884 | -0.051 | -0.2791 | No |
| 36 | LY6E | na |  |  | 11120 | -0.081 | -0.2901 | No |
| 37 | HLA-C | na |  |  | 11355 | -0.097 | -0.3008 | No |
| 38 | IRF2 | na |  |  | 12188 | -0.198 | -0.3411 | No |
| 39 | LGALS3BP | na |  |  | 12273 | -0.209 | -0.3420 | No |
| 40 | GBP4 | na |  |  | 12911 | -0.273 | -0.3709 | No |
| 41 | TRIM21 | na |  |  | 12921 | -0.274 | -0.3669 | No |
| 42 | CD74 | na |  |  | 12971 | -0.280 | -0.3649 | No |
| 43 | CXCL11 | na |  |  | 13037 | -0.289 | -0.3636 | No |
| 44 | TMEM140 | na |  |  | 13078 | -0.296 | -0.3608 | No |
| 45 | IFIH1 | na |  |  | 13280 | -0.322 | -0.3661 | No |
| 46 | IRF9 | na |  |  | 13588 | -0.367 | -0.3761 | No |
| 47 | ISG20 | na |  |  | 13723 | -0.391 | -0.3767 | Yes |
| 48 | TRIM5 | na |  |  | 13740 | -0.393 | -0.3711 | Yes |
| 49 | PNPT1 | na |  |  | 13873 | -0.414 | -0.3713 | Yes |
| 50 | IFITM1 | na |  |  | 14071 | -0.427 | -0.3746 | Yes |
| 51 | NMI | na |  |  | 14136 | -0.432 | -0.3709 | Yes |
| 52 | GMPR | na |  |  | 14205 | -0.442 | -0.3672 | Yes |
| 53 | DDX60 | na |  |  | 14208 | -0.443 | -0.3600 | Yes |
| 54 | FAM46A | na |  |  | 14257 | -0.454 | -0.3551 | Yes |
| 55 | GBP2 | na |  |  | 14345 | -0.470 | -0.3520 | Yes |
| 56 | TRAFD1 | na |  |  | 14581 | -0.503 | -0.3561 | Yes |
| 57 | PROCR | na |  |  | 14847 | -0.543 | -0.3610 | Yes |
| 58 | RSAD2 | na |  |  | 14852 | -0.544 | -0.3523 | Yes |
| 59 | RTP4 | na |  |  | 14854 | -0.544 | -0.3435 | Yes |
| 60 | PSMA3 | na |  |  | 15032 | -0.567 | -0.3435 | Yes |
| 61 | IL15 | na |  |  | 15098 | -0.579 | -0.3374 | Yes |
| 62 | PSME2 | na |  |  | 15141 | -0.587 | -0.3300 | Yes |
| 63 | BST2 | na |  |  | 15210 | -0.597 | -0.3238 | Yes |
| 64 | NUB1 | na |  |  | 15338 | -0.624 | -0.3202 | Yes |
| 65 | PSMB8 | na |  |  | 15412 | -0.636 | -0.3136 | Yes |
| 66 | UBE2L6 | na |  |  | 15591 | -0.666 | -0.3121 | Yes |
| 67 | LAP3 | na |  |  | 15631 | -0.674 | -0.3031 | Yes |
| 68 | PSMB9 | na |  |  | 15703 | -0.682 | -0.2956 | Yes |
| 69 | PARP9 | na |  |  | 15934 | -0.730 | -0.2957 | Yes |
| 70 | IFITM3 | na |  |  | 15996 | -0.744 | -0.2867 | Yes |
| 71 | NCOA7 | na |  |  | 16118 | -0.772 | -0.2804 | Yes |
| 72 | EIF2AK2 | na |  |  | 16137 | -0.775 | -0.2687 | Yes |
| 73 | WARS | na |  |  | 16504 | -0.855 | -0.2739 | Yes |
| 74 | RIPK2 | na |  |  | 16616 | -0.878 | -0.2653 | Yes |
| 75 | IFITM2 | na |  |  | 16644 | -0.884 | -0.2522 | Yes |
| 76 | SAMD9L | na |  |  | 16828 | -0.934 | -0.2465 | Yes |
| 77 | IFI44 | na |  |  | 16919 | -0.962 | -0.2355 | Yes |
| 78 | TDRD7 | na |  |  | 16953 | -0.970 | -0.2214 | Yes |
| 79 | BATF2 | na |  |  | 17399 | -1.118 | -0.2264 | Yes |
| 80 | PLSCR1 | na |  |  | 17416 | -1.120 | -0.2089 | Yes |
| 81 | TXNIP | na |  |  | 17600 | -1.197 | -0.1989 | Yes |
| 82 | PSME1 | na |  |  | 17774 | -1.276 | -0.1871 | Yes |
| 83 | IFI27 | na |  |  | 17788 | -1.281 | -0.1668 | Yes |
| 84 | ELF1 | na |  |  | 17853 | -1.311 | -0.1487 | Yes |
| 85 | USP18 | na |  |  | 17930 | -1.341 | -0.1307 | Yes |
| 86 | IFIT2 | na |  |  | 18078 | -1.429 | -0.1150 | Yes |
| 87 | IFIT3 | na |  |  | 18445 | -1.669 | -0.1068 | Yes |
| 88 | CD47 | na |  |  | 18753 | -1.956 | -0.0909 | Yes |
| 89 | SAMD9 | na |  |  | 18907 | -2.175 | -0.0633 | Yes |
| 90 | CCRL2 | na |  |  | 18912 | -2.189 | -0.0277 | Yes |
| 91 | B2M | na |  |  | 19069 | -2.604 | 0.0068 | Yes |
Table: GSEA details [plain text format]

  

Fig 2: HALLMARK\_INTERFERON\_ALPHA\_RESPONSE      
 Blue-Pink O' Gram in the Space of the Analyzed GeneSet

  

Fig 3: HALLMARK\_INTERFERON\_ALPHA\_RESPONSE: Random ES distribution      
 Gene set null distribution of ES for **HALLMARK\_INTERFERON\_ALPHA\_RESPONSE**
